# A Novel S100A8/A9 Induced Fingerprint of Mesenchymal Stem Cells associated with Enhanced Wound Healing

**DOI:** 10.1038/s41598-018-24425-9

**Published:** 2018-04-18

**Authors:** Abhijit Basu, Saira Munir, Medanie A. Mulaw, Karmveer Singh, Diana Crisan, Anca Sindrilaru, Nicolai Treiber, Meinhard Wlaschek, Markus Huber-Lang, Florian Gebhard, Karin Scharffetter-Kochanek

**Affiliations:** 1 Experimental Laboratories of the Department of Dermatology and Allergic Diseases, Ulm University, Life Science Building N27, James-Franck-Ring, 89081 Ulm, Germany; 2 Institute of Experimental Cancer Research, Ulm University, Life Science Building - N27, James-Franck-Ring, 89081 Ulm, Germany; 3 Department of Dermatology and Allergic Diseases, Ulm University, Albert-Einstein-Allee 23, 89081 Ulm, Germany; 4 Institute for Clinical and Experimental Trauma Immunology (ITI), Ulm University, Helmholtzstraße 8/2, 89081 Ulm, Germany; 5 Department of Orthopaedic Trauma-, Hand-, Plastic, and Reconstruction Surgery, Ulm University, Albert-Einstein-Allee 23, 89081 Ulm, Germany

## Abstract

We here investigated whether the unique capacity of mesenchymal stem cells (MSCs) to re-establish tissue homeostasis depends on their potential to sense danger associated molecular pattern (DAMP) and to mount an adaptive response in the interest of tissue repair. Unexpectedly, after injection of MSCs which had been pretreated with the calcium-binding DAMP protein S100A8/A9 into murine full-thickness wounds, we observed a significant acceleration of healing even exceeding that of non-treated MSCs. This correlates with a fundamental reprogramming of the transcriptome in S100A8/A9 treated MSCs as deduced from RNA-seq analysis and its validation. A network of genes involved in proteolysis, macrophage phagocytosis, and inflammation control profoundly contribute to the clean-up of the wound site. In parallel, miR582-5p and genes boosting energy and encoding specific extracellular matrix proteins are reminiscent of scar-reduced tissue repair. This unprecedented finding holds substantial promise to refine current MSC-based therapies for difficult-to-treat wounds and fibrotic conditions.

## Introduction

Wound healing comprises a complex, highly regulated sequence of different molecular and cellular events with the ultimate goal to functionally restore tissue damage after trauma^1–4^.

The different healing phases are partly overlapping and involve the clotting phase with a first provisional closure of wounds.Thereafter the inflammatory phase occurs to fight against bacteria and to clean wound debris by the attraction of neutrophils and monocytes and debris phagocytosis.The inflammatory phase is followed by the phase of granulation tissue formation with angiogenesis and the contraction of wounds by α-smooth muscle actin positive myofibroblasts. Finally, the phase of matrix synthesis with deposition of collagenous and non-collagenous proteins and their subsequent remodeling takes place. Wound healing in mammals and humans results in scar formation with a partial to complete lack of regeneration of skin appendages like sweat glands and hairs^5,6^

Mesenchymal stem cells have earlier been reported to coordinate histogenetically distinct cell species involved in different phases during wound healing in a variety of preclinical murine models^7–9^ and in impaired human wounds^10–14^ leading to accelerated and scar reduced tissue repair. Due to these beneficial effects, MSC-based therapies are currently assessed in clinical phase I/IIa studies to improve wound healing with accelerated wound closure, suppression of inflammation and scar reduced healing^13,15,16^.

Though not studied in detail, MSCs are endowed with the capacity to sense their environment at the wound site and to mount mainly paracrine effector responses which impact on different cell species involved in inflammation, angiogenesis, re-epithelialization, wound contraction and deposition of extracellular matrix^7–9^. During tissue trauma, endogenous MSCs or MSCs applied to the wound site in a therapeutic intent are exposed to a variety of cues which impact on their expression signature. Among growth factors, cytokines, extracellular matrix molecules, pathogen-associated molecular patterns (PAMPs) and danger associated molecular patterns (DAMPs) constitute an ever-changing microenvironment at the wound site. Recently, the calcium binding protein complex S100A8/A9, a DAMP molecule occurring at the site after severe tissue trauma was reported to play a crucial role in the repair of renal injury^17^, and revealed a strong decrease in chronic non-healing wounds in humans^18^.

S100A8/A9 can be recognized by the Toll-like receptor 4 which is expressed on MSCs. Even though the impact of various DAMPs on MSCs, mainly on their proliferative and migratory responses has been reported^9,19,20^, the impact of S1008/A9 on the adaptive transcriptome response on MSCs have never been addressed in the context of wound healing. We here show that MSCs pretreated with S100A8/A9 prior to being injected at the wound site substantially increased wound healing in a murine full thickness wound model. To further understand this beneficial effect, we set out for an unbiased comprehensive RNAseq analysis. We uncovered a previously unreported transcriptome signature of S100A8/A9 treated MSCs which includes genes involved in cleaning up the wound site by control of proteolytic enzymes like serpins and their inhibitors, activation of complement factors promoting macrophage engulfment of tissue debris.In addition, genes induced by S100A8/A9 priming of MSCs impacts on MSC recruitment as shown previously to resolve inflammation and to enhance angiogenesis. Enhanced synthesis of the glycoprotein SPARC and suppression of thrombospondin 7 in S100A8/A9 primed MSCs contribute to a transient restoration tissue most likely with scar reduced healing. In addition, our data show that energy homeostasis is boosted and a beneficial niche supporting stem cells and resident cells are implemented. We feel that this novel transcriptome signature constitute a valuable resource for researchers interested in DAMP effects on MSCs and in refining MSC-based therapies for tissue repair.

## Results

### MSCs pretreated with S100A8/A9 induced accelerated healing of murine wounds

**Figure 1:**
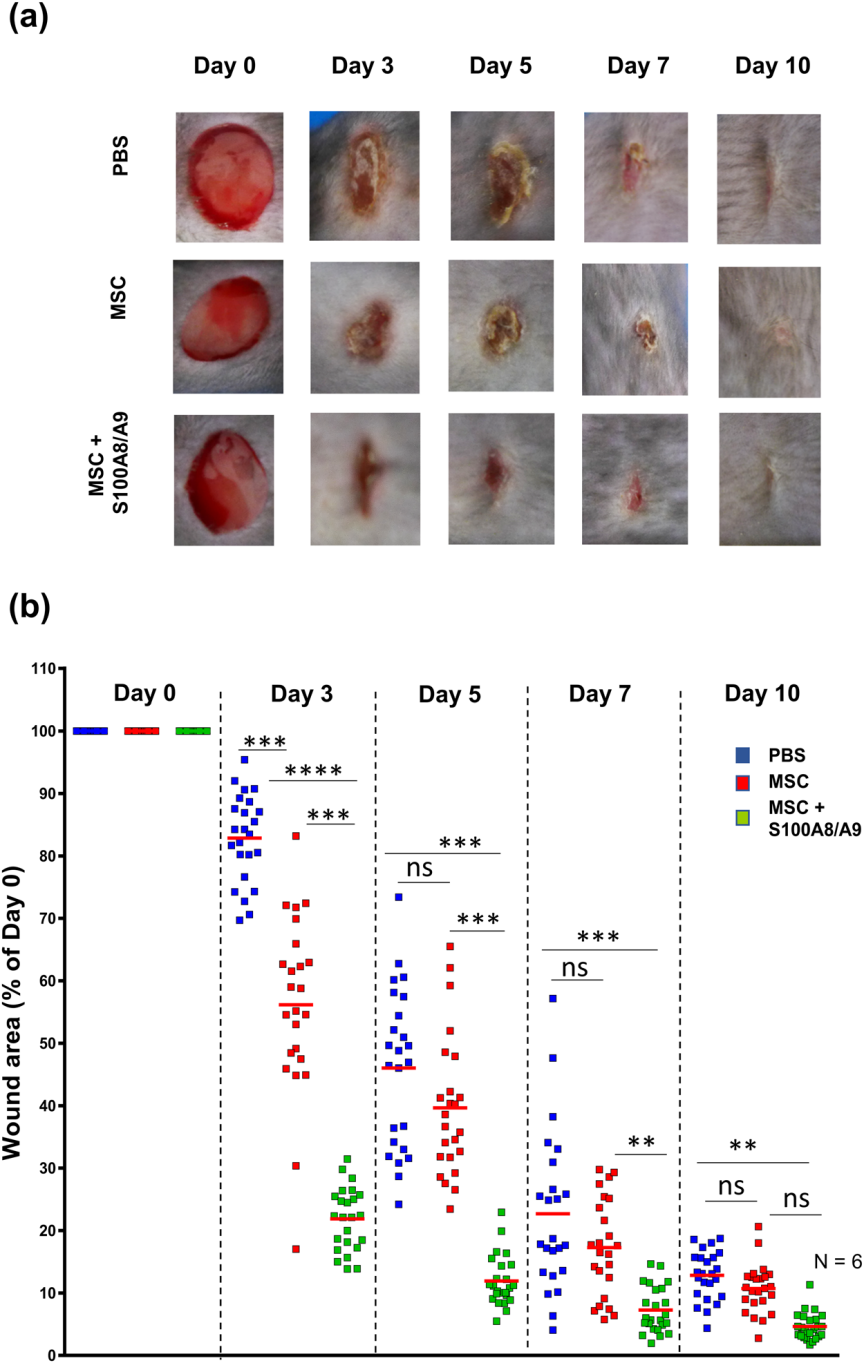
S100A8/A9 activated MSCs significantly accelerate wound healing. Full-thickness excisional wounds including the panniculus carnosus were produced on the back of mice by 6 mm biopsy round knives. Wounds margins were intradermally injected with PBS or with 2.5 × 10^5^ MSCs (either non-treated or treated with S100A8/A9) or PBS. Each wound was digitally photographed at the indicated time points, and wound areas were analyzed using Adobe Photoshop. (**a**) Representative macroscopic pictures of PBS or MSC or MSC treated with S100A8/A9 wounds at day 0 and 3, 5, 7 and 10-day post-wounding. (**b**) Quantitative analysis of 24 wound areas per group, representing wound closure as percentage of the initial wound size at day 0. Blue symbols represent wound sizes of the PBS treated group, red symbols represent wound sizes following intradermal MSC injection and green symbols represent wound sizes following injection with MSCs pre-activated with 0.5 µg/mL Rh S100A8/A9 protein. The red line in each group represents the mean value of 24 wounds from 6 mice. The significance among the groups were calculated using ANOVA with non-parametric measures using Kruskal Wallis and Dunns’ post hoc test where *p < 0.05, **p < 0.01, ***p < 0.001, ****p < 0.0001 ns, non-significant.

To assess whether MSCs effector functions change in response to the DAMP molecule A8/A9 and, in consequence, accelerates wound closure, fully characterized MSCs (Supplementary Fig. 1) have been subjected to recombinant human S100A8/A9 at a concentration of 5 µg/ml prior to being injected around 6 mm full thickness wounds. Wound closure was digitally assessed and compared to PBS injected wounds and to wounds which were injected with non-treated control MSCs. Interestingly, wound closure was significantly accelerated in wounds injected with S100A8/A9 pretreated MSCs as opposed to wounds injected with non-treated control MSCs at day 3 and 5 (p > 0.0001) and day 7 (p > 0.001) (Fig. 1). This is remarkable as injection of control MSCs already resulted in enhanced wound closure in initial wound healing when compared to PBS injected control wounds. These data very much suggest that pretreatment of MSCs with S100A8/A9 result in reprogramming of the transcriptome and this is associated with a remarkably enhanced wound closure.

### MSCs pretreated with S100A8/A9 reveal a distinct transcriptome signature with impact on enhanced tissue repair

**Figure 2:**
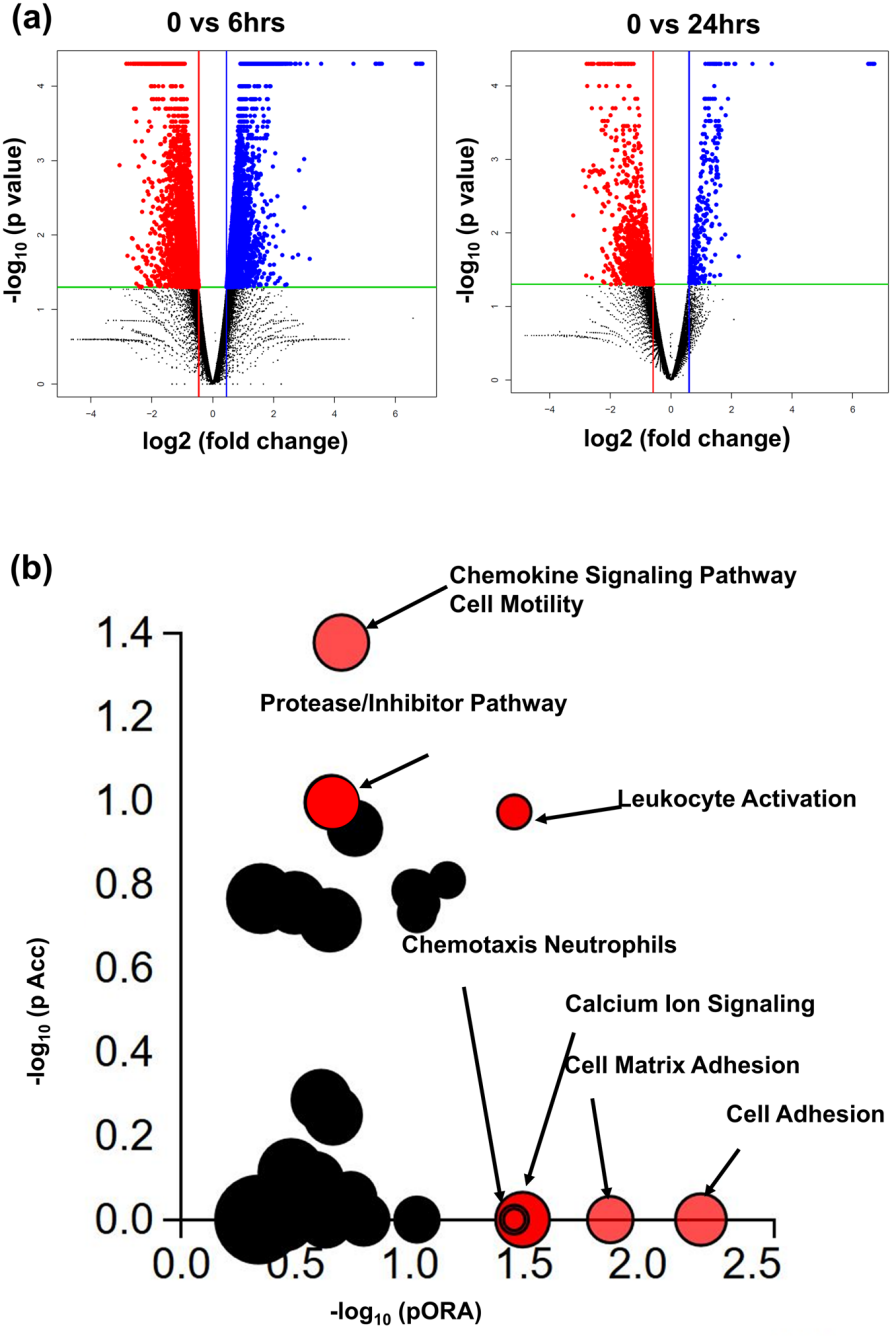
MSCs pretreated with S100A8/A9 reveal a distinct transcriptome signature with impact on enhanced tissue repair. **(a**) Volcano plot representative of the entire gene expression data (untreated MSCs versus MSCs treated with S100A8/A9 for 6 h (left) and 24 h (right) were studied by DEseq. 2 analysis tool, where the log_10_ P-value (FDR <0.5) of each gene is plotted against log _2_ fold for that gene. Differentially expressed (DE) genes (FDR <5%) and fold change (mean >1.5) are highlighted either in blue as upregulated or highlighted in red as downregulated. The non-significant genes which (FDR >5%) are highlighted in black. (**b**) Pathway analysis was done on the DE genes (log_2_-fold change >0.6 and FDR <5%) employing iPathwayGuide analysis tool that uses two types of evidence: the over-representation on the horizontal axis (pORA) and the perturbation on the vertical axis (pAcc). Significant pathways with (FDR <5%) are shown in red, whereas non-significant are in black. The size of the circle is proportional to the number of genes in that pathway.

To identify genes which may enforce enhanced wound closure, RNA-seq was performed. First, genes differentially expressed between control MSCs and MSCs treated with S100A8/A9 were compared. Figure 2a,b shows the distribution of log_2_-fold change of 0.6 and adjusted p-value < 0.05 for genes expressed at 6 and 24 h after treatment of MSCs with S100A87A9 as compared to non-treated MSCs. The differentially expressed genes between control MSCs and S100A8/A9 are highlighted in blue (up-regulated genes) and in red (down-regulated genes). Among differentially expressed genes 4848 and 312 were up-regulated during 6 and 24 h, and 5217 and 1700 were down-regulated in S100A8/A9 treated MSCs during 6 and 24 h. To further study the pathways which are different in S100A8/A9 treated MSCs compared to control MSCs, Impact analysis was performed using iPathwayGuide and the Gene trail tool (Fig. 2c). Seven major pathways were uncovered in S100A8/A9 treated MSCs using topological analysis. The most significant pathways included cell motility, cell/leukocyte activation and chemotaxis, chemokine signaling, protease control, calcium signaling and cell-matrix adhesion.

**Figure 3:**
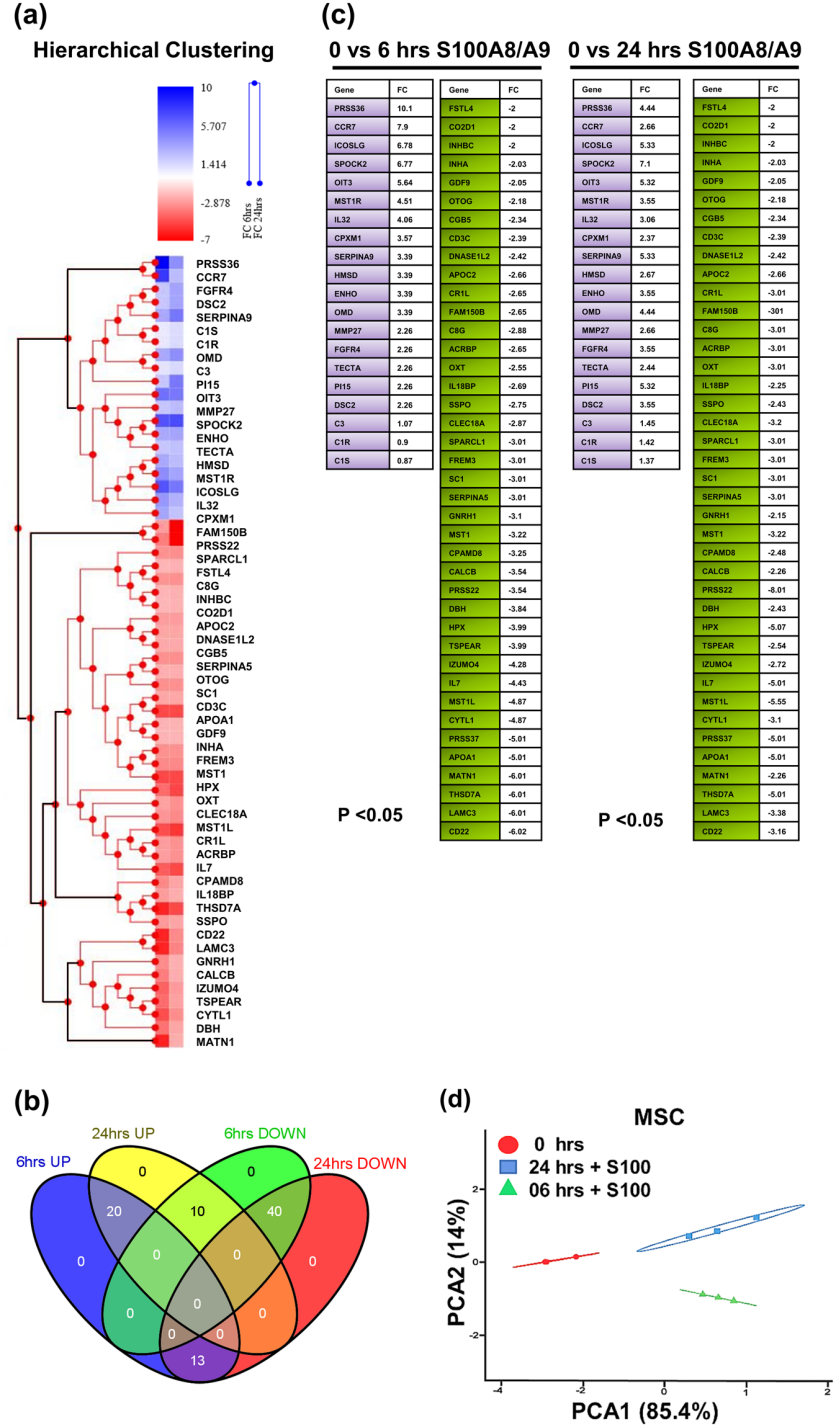
RNAseq expression profile of MSCs exposed to S100A8/A9. **(a**) Shows the hierarchical clustering of pre-ranked secretory genes using Pearson’s correlation for the RNAseq expression profile of human MSCs stimulated with S100A8/A9 for 6 and 24 h as compared to PBS treated control MSCs. The upper left panel shows the fold changes (FC) of gene expression after 6 and after 24 h of MSC treatment with S100A8/A9 compared to PBS treated MSCs. Blue shades show an increase in gene expression, red shades show a decrease in gene expression as compared to PBS treated control MSCs. The sub clusters below depict genes which are either persistently up-regulated or downregulated at 6 and at 24 h following S100A8/A9 exposure. **(b)** The Venn diagram highlight the overlap of genes which are up-regulated or down regulated at 6 and 24 h. We specifically concentrated on the expression profile of genes which are persistently up-regulated at 6 and 24 h (20 genes in light purple color) or persistently down-regulated at 6 and 24 h after S100A8/A9 exposure (40 genes in shaded green). **(c**) Depicts the expression levels (as fold change) and the annotation of the corresponding genes. (**d**) The first principal component accounted for 85.4% of the total variability, which in this case corresponds to the unstimulated MSC variance (highlighted in red), and the subsequent principal components accounted for 14% of overall variance (highlighted in green & blue), highlighting the difference between the unstimulated MSC and S100A8/A9 stimulated MSC (6 h and 24 h), respectively. Principal component analysis (PCA) of the sample correlation matrix calculated from log_2_ transformed FPKM values. Differences in gene expression of at least 2-fold and a P-value < 0.05 (Student´s T-Test) were selected.

Hierarchical clustering analysis of the pre-ranked gene-based Pearson correlation for the RNAseq expression profile of human MSCs stimulated with S100A8/A9 for 6 and 24 hr as compared to PBS treated control MSCs (Fig. 3a) The Venn diagram depicts the overlap of pre-ranked differentially expressed genes of the extracellular secretory pathway which are up-regulated or down-regulated at 6 h and 24 h (Fig. 3b). We specifically focused on the expression profile of genes which are persistently up-regulated at 6 and 24 h (20 genes) or persistently down-regulated at 6 and 24 h after S100A8/A9 exposure (40 genes). These selected genes are shown in Fig. 3c with the up-regulated genes in light purple and the down-regulated genes in shaded green. Many of these genes are in line with the preceding pathway analysis (Fig. 2b). The Principal Component Analysis (PCA) was carried out using regularized log transformed FPKM values with an adjusted p-value < 0.05. The first principal component accounted for 85.4% of the total variability, which in this case corresponds to the unstimulated MSC variance (shown in red), and the subsequent principal components accounted for 14% of overall variance (shown in green & blue), highlighting the difference between the unstimulated MSC and S100A8/A9 stimulated MSC (6 h and 24 h), respectively.

#### Cell Motility and cell/leukocyte activation

Several genes which are highly up-regulated in S100A8/A9 treated MSCs are involved in the recruitment of neutrophils, macrophages and endogenous MSCs to the wound site (IL-32, CR3a, CCR7). Also, genes involved in leukocyte activation enhancing their killing ability and phagocytic properties and genes involved in the downregulation of T cell effector functions are up-regulated in S100A8/A9 treated MSCs (IL-32, C1R, MSTR1, C3, CFB, B2, CFB, CFD, ICOSLG, CD22). MSCs express several chemokine receptors among them **CCR7**, which by binding to its ligand CCL21 enhances MSC migration. Of note, RNAseq analysis showed CCR7 to be enhanced at 6 and 24 h after exposure of MSCs to S100A8/A9. In fact, previously it was reported that intradermal injection of CCL21 increased MSC migration to the wound site^21^. Of note, injured epidermis releases CCL21 thus ensuring that MSCs are directed to the site of skin damage^22^. Specific mRNA for the MST1 receptor (MST1R) was increased 4.51-fold at 6 h and increased 2.55 - fold at 24 h after MSC treatment with S100A8/A9. The **MST1R gene** codes for the macrophage stimulating 1 receptor which upon binding to its ligand, the macrophage-stimulating protein (MSP), reveals tyrosine kinase activity and enhances macrophage recruitment to the wound site and their phagocytotic activity. Similarly, it may enhance MSC phagocytosis. In fact, we recently showed that MSCs can phagocytose neutrophils and thus contribute to clearance of the wound site and resolution of the inflammatory phase. It is 4.06 - fold increased at 6 h and 3.06 - fold increased at 24 h after MSC exposure to S100A8/A9. The expression of the **ICOSLG gene** coding for the ligand for the T-cell-specific cell surface receptor ICOS was increased 6.78 - fold at 6 h and 5.38 - fold at 24 h after exposure of MSCs to S100A8/A9. ICOSLG is involved in regulatory T-cell dependent suppression of effector T cells and acts as a costimulatory signal for regulatory T-cell proliferation^23,24^, cytokine secretion^25^ among other functions. Also, the **OIT3 gene** encodes for the oncogene induced transcript 3 protein which is reported to be liver specifically expressed and plays a role in development. This gene was significantly increased in MSCs after S100A8/A9 exposure. The role of OIT3 is currently unclear, but it may enhance protection (also see discussion). The **ENHO** (Energy Homeostasis Associated) is a protein-coding gene. It is involved in the regulation of glucose homeostasis and lipid metabolism (for further information see discussion).

#### Proteases and their inhibitors

We uncovered an enhanced enrichment score of 2.52 with an up to 9-fold increase in genes encoding the *serine-type endopeptidase and serine hydrolase* expression accompanied with enrichment scores of 2.35 an up to 7-fold increase in *the corresponding inhibitors* suggesting an enhanced though controlled clean up tissue debris at the wound site (Fig. 2b). Genes clustered in this group include **PRSS36** coding for polyserase-2, a secreted serine protease whose expression is increased in S100A8/A9 treated MSCs at 6 h after treatment 10.1-fold and 24 h after treatment still 4.4-fold. Also **SERPINA9**, a serine protease inhibitor involved in inhibiting serine proteases revealed an increase in expression of 3.39 at 6 h and 5.0 at 24 h after exposure to S100A8/A9. Similarly, the **HMSD gene** encoding for a serpin-domain containing protein that functions as a serine protease inhibitor was up-regulated 3.39-fold after 6 h and 5.5-fold after 24 h of S100A8/A9 treatment of MSCs. An increase of 2.26 after 6 h and 5.39 after 24 h of S100A8/A9 of specific Pi15 mRNA was found. The **Pi15 gene** codes another serine protease inhibitor which displays weak inhibitory activity against trypsin. Apart from the controlled balance of serine proteases and their inhibitors, **MMP-27**, a family member of the matrix-degrading metalloproteinases was up-regulated 2.26 - fold after 6 h and 2.66 - fold after 24 h of MSCs treatment with S100A8/A9.

#### Calcium signaling and cell-matrix adhesion

Also, calcium signaling-related genes and pathways reveal a strong enrichment score of 2.35 and an up to 3.3 fold change in gene expression of S100A8/A9 pretreated MSCs as opposed to untreated MSCs. Accordingly, the **Spock-2 gene** encodes a glycoprotein (SPARC, BM-40, Osteonectin) which binds to glycosaminoglycans as an important core component of proteoglycans in the extracellular matrix. After exposure of MSCs to S100A8/A9 Spock-2 is up-regulated 6.77- fold at 6 h and 7.10 -fold at 24 h. The **TECTA gene** codes for α-tectorin, a major non-collagenous connective tissue component of the tectorial membrane. The **Dsc2 gene** encodes for Desmocollin-2, a calcium-dependent cadherin-type protein, that functions to link adjacent cells together in specialized regions known as desmosomes, but has not been described in MSCs.

### Identification of miR-582-5p involved in the S100A8/A9 induced MSC fingerprint

**Figure 4:**
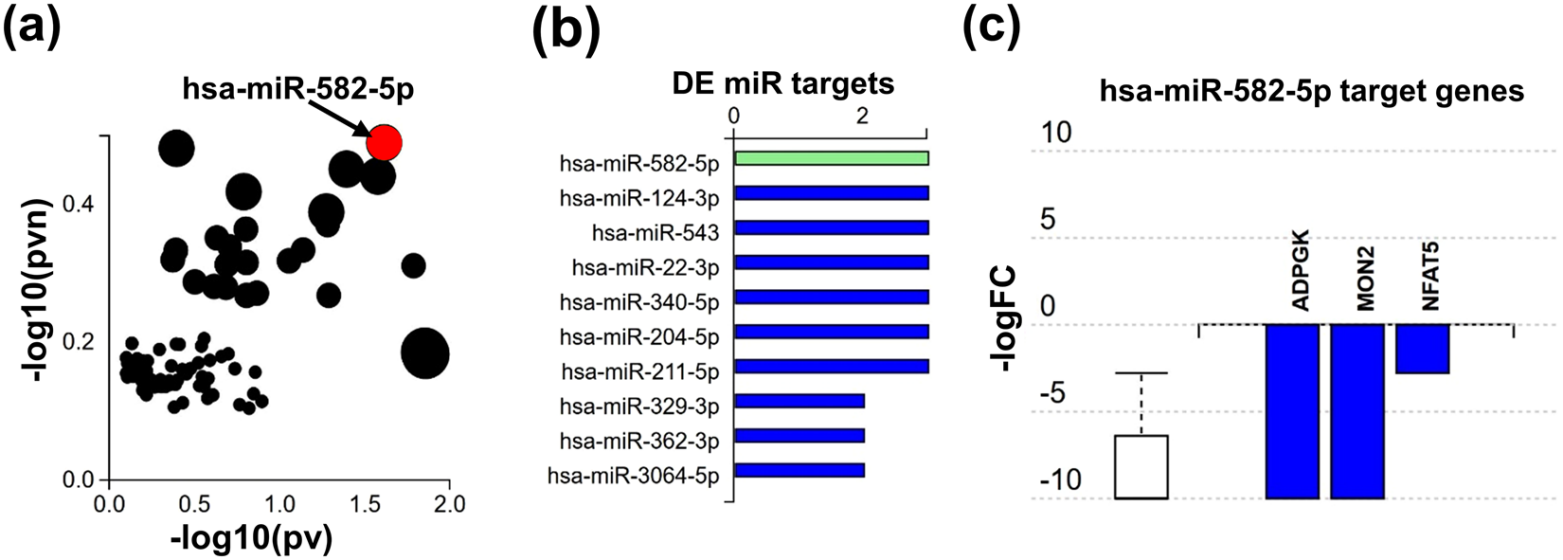
Differential miR expression and target genes between S100A8/A9 treated MSCs and control MSCs. (**a**) Significant differences in specific miR expression. Analysis is based on the differentially expressed (DE) miR (log_2_-fold change >0.6 and FDR <5%) using iPathwayGuide analysis tool that uses two types of evidence to represent the X-Y plot: the horizontal axis (−log_10_(pv)) representing the p-value based on the total number of DE target genes versus the total number of target genes and the vertical axis the (−log_10_(pvn)) is the p-value based on the number of downwardly expressed DE targets versus the total number of DE miR targets. The size of each dot is directly proportional to number of target genes for that miRNA relative to other ones. Significant miR’s with (FDR <5%) such as has-miR 582-5p are shown in red, whereas non-significant are in black. (**b**) Represents the miR expression profile of non-stimulated versus stimulated MSCs with S100A8/A9 depicting the relative counts for each miRNA. **(c)** Shows selected **miRNA** hsa-miR-582-5p target genes which are significantly suppressed following S100A8/A9 treatment of MSCs. The suppression is represented by log fold change in stimulated MSCs versus non-stimulated MSCs.

Specific miR expression was assessed in an unbiased global approach. Only the hsa-miR-582 reached significant expression values as depicted in Fig. 4a,b. Figure 4c displays target genes which expression is significantly suppressed by has-miR-582-5p. Among them, the ADPGK gene coding for the human ADP-dependent glucokinase which is involved in glycolysis^26^. Suppression of glycolysis may indicate that MSCs after exposure to S100A8/A9 switch their high metabolic demands during tissue repair to faster and more effective energy-generating pathways. Also, the human MON2 gene encoding a protein which controls the traffic between endosomes and the Golgi apparatus^24^ is suppressed by S100A8/A9 induced miR-582-5p. MON2 suppression may also help to save energy urgently required for wound site debridement, extracellular matrix synthesis, angiogenesis, and re-epithelialization. Finally, the NFAT5 gene encoding the transcription factor NFAT5 is significantly reduced by the S100A8/A9 induced miR-582-5p. This most likely is relevant in terms of energy homeostasis. NFAT5 induces osteogenic differentiation of MSCs^27^ which, in fact, would demand a lot of energy, while it is distinctly counterproductive for cutaneous tissue repair.

### Validation of RNA-seq data via qRT-PCR

To validate the RNA-seq data, expression levels of selected differentially regulated genes are validated by qRT-PCR. For this purpose, RNA was isolated from S100A8/A9 treated MSCs and from control MSCs. Our RNA-seq data show that pathways essential for tissue repair are mainly impacted following MSC exposure to S100A8/A9.

**Figure 5:**
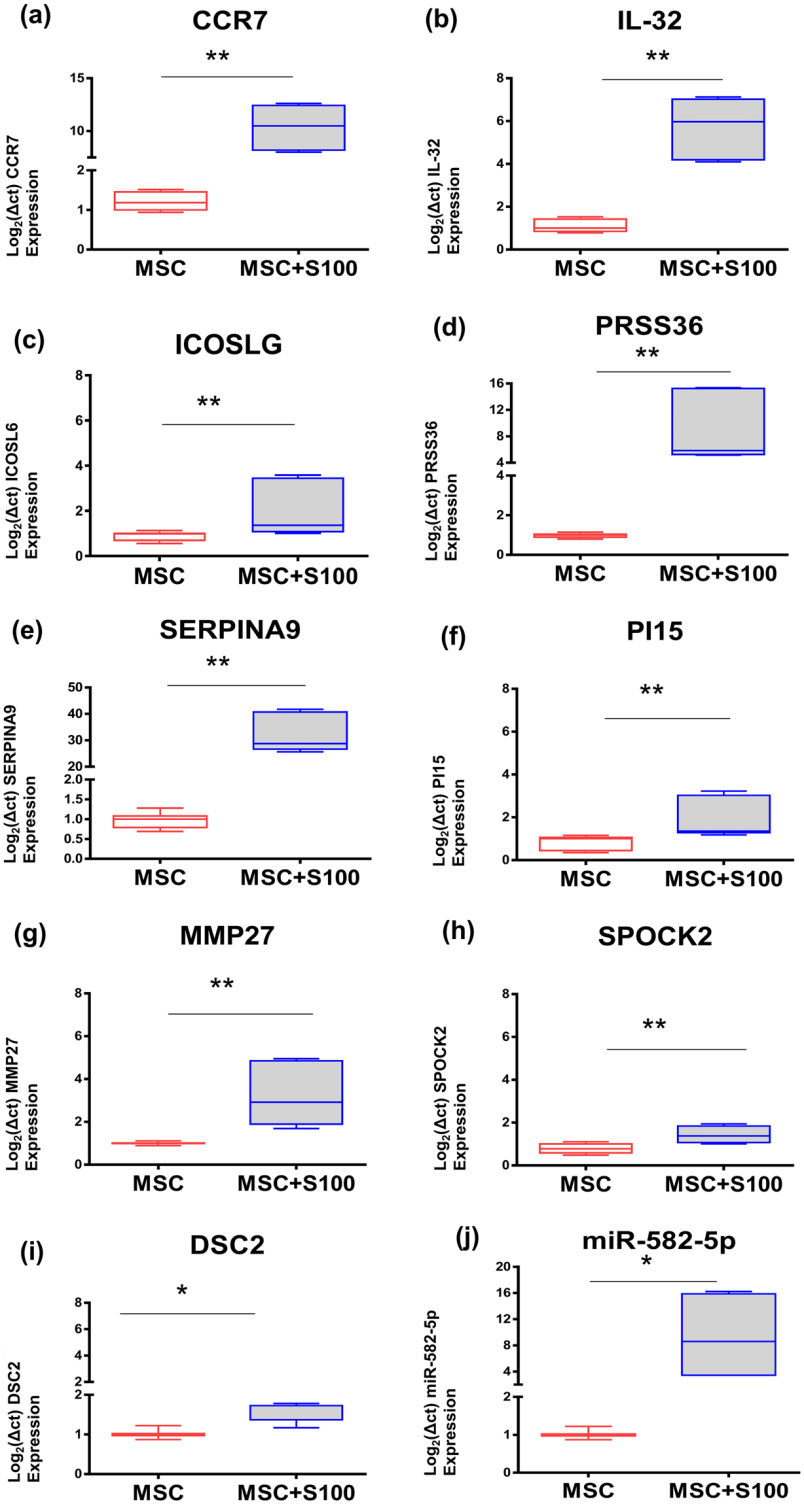
Validation of RNA-seq data by qRT-PCR. Total RNA and miRNA was extracted from control MSCs and from MSCs which have been treated with 0.5 µg/mL S100A8/A9. Isolated RNAs were assessed for quality with Bioanalyzer and reverse-transcribed using the Reverse Transcription System. The primers targeted **(a)** CCR7, **(b)** IL-32, **(c)** ICOSLG, **(d)** PRSS36, **(e)** SERPINA9, **(f)** PI15, (**g**) MMP27, **(h)** SPOCK2, (**i**) DSC2, (**j**) hsa-miR-582-5p, to amplify cDNA using 2x power SYBR Green Mix (Applied Biosystems). Relative mRNA or miR levels were calculated by normalizing to β-actin and RNU6B in case of miRNA. Box plots are expressed as mean with min-max wiskers representing the normalized expression level of each gene of MSCs treated with S100A8/A9 as opposed to control MSCs. Data were analyzed using the unpaired, two-tailed non-parametric Student’s t test using Wilcoxon signed rank-test and the differential expression of the qPCR results were expressed as log_2_ fold change with the respective p-values. P-value < 0.05 and less were considered significant.

Among the differentially expressed genes involved in cell motility and recruitment to the wound site, we chose the gene encoding the chemokine receptor CCR7 which mediates MSC recruitment to the wound site (Fig. 5a). In addition, the up-regulation of IL-32 involved in neutrophil as well as macrophage recruitment and re-epithelialization was confirmed by qRT-PCR (Fig. 5b). Induced ICOSLG gene expression coding for the ligand which stimulates regulatory T cells as found by RNAseq analysis was confirmed by qRT-PCR (Fig. 5c). This, in consequence, contributes to the overall resolution of the T cell-mediated inflammatory response. This is important as T cells via Interferon maintain macrophages in an unrestrained activation state^28^. We selected 4 genes within the enriched proteases/inhibitors pathway. These include PRSS36 coding for a serine protease (Fig. 5d), SERPINA9 coding for a serine protease inhibitor (Fig. 5e) and, similarly, Pi15 coding for a serine protease inhibitor (Fig. 5f). In addition, MMP-27, a matrix-metalloprotease with cleavage specificity for extracellular matrix proteins was selected (Fig. 5g). All were confirmed to be induced following exposure of MSCs to S100A8/A9. Furthermore, we chose the SPOCK-2 gene encoding SPARC (BM80; Osteonectin) within the calcium signaling and matrix adhesion pathway. SPARC is a glycoprotein regulated by calcium fluxes and previously reported to be essential in tissue regeneration and developmental patterning. It mediates cell-matrix interactions and similar to RNAseq data SPOCK-2 expression was confirmed to be highly up-regulated by qRT-PCR (Fig. 5h). Expression of the DSC2 gene coding for Desmocollin 2 was confirmed to be up-regulated in S100 A8/A9 treated MSCs (Fig. 5i). Of note, also miR582-5p was up-regulated by qRT-PCR (Fig. 5i).

### Validation of the MSC S100A8/A9 fingerprint *in vivo*

**Figure 6:**
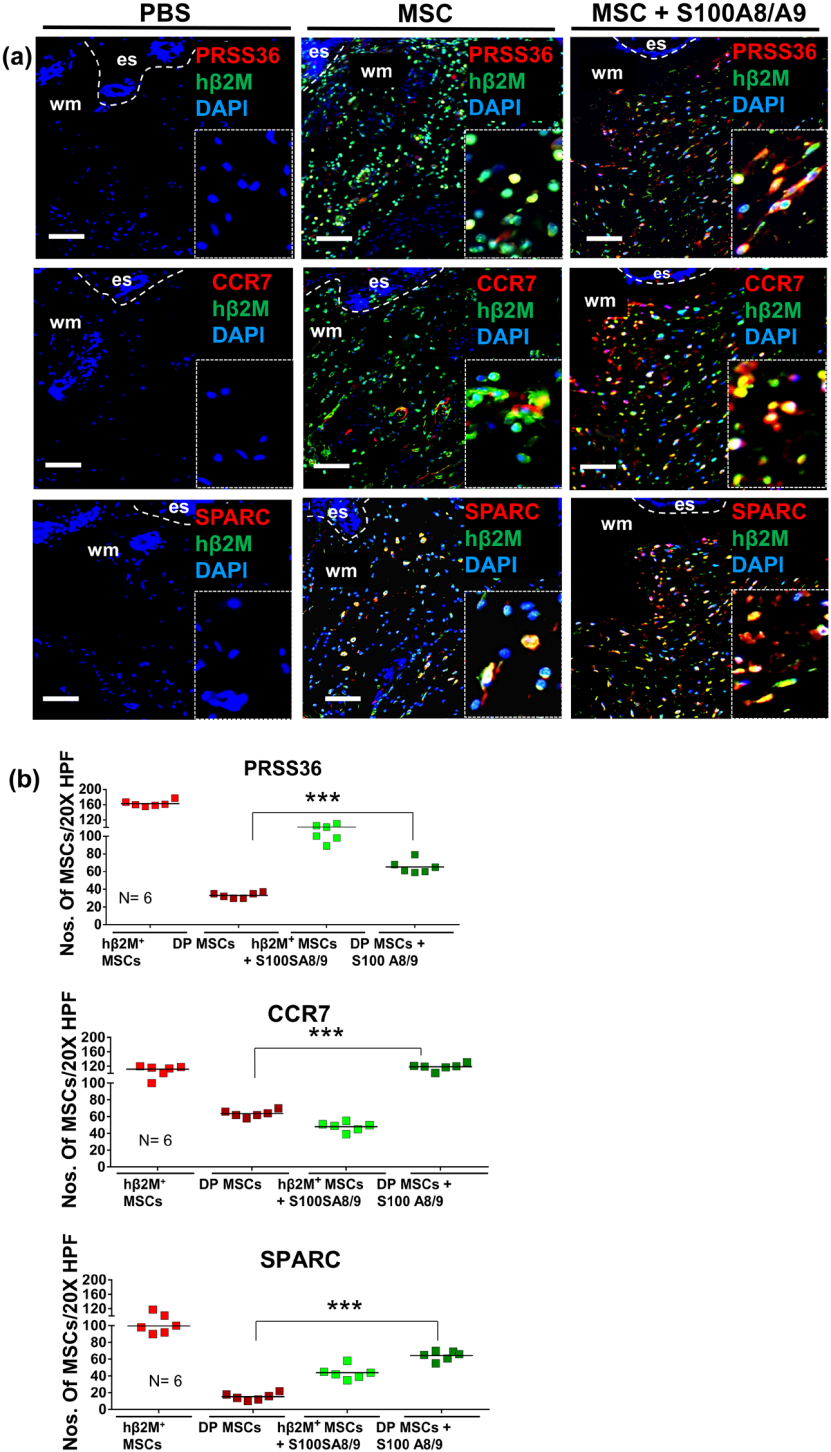
Immunostaining for selected proteins encoded by up-regulated genes after injection of S100A8/A9 treated MSCs into wounds. Representative photomicrographs of sections from wounds which were injected with either control MSCs or S100A8/A9 treated MSCs. The expression of selected genes on protein level is shown with immunostaining for the serine protease PRSS36, the MSC specific chemokine receptor CCR7, and the developmental-like proteoglycan SPARC. Double staining (DP) for human β2 microglobulin (hβ2M is shown in green detecting the injected MSCs) and PRSS36 (shown in red) **(a)**; CCR7 (shown in red) **(b)** and SPARC (shown in red) was compared to single staining with hβ2M. Nuclei were counterstained with DAPI. Scale bars: 200 μm. Dashed lines indicate the junction between epidermis (e) and dermis. es, eschar; wm, wound margin. Quantification of double-stained and single hβ2M^+^ MSCs in wounds injected with control MSCs and S100A8/A9 treated MSCs at days 2 after wounding were counted in 10 high-power fields per sample. Results are mean ± SD ratio of DP + and single hβ2M^+^ MSCs to total cells counted in the dermis (n = 6). ***P < 0.0001 versus non-treated control MSC injected wounds and MSCs pretreated with S100A8/A9 prior to be injected into wounds. The significance was obtained using ANOVA with Tukeys post hoc test.

Previously, we have shown that >70% of MSCs are still present at the wound site at 24 hrs after MSCs injection to the wound site^29^. To explore whether the S100A8/A9 fingerprint observed in MSCs *in vitro* may also play a role *in vivo*, we stained sections from wounds which 24 h before had been injected with S100A8/A9 treated MSCs (Fig. 6). Human β2 microglobulin served as a marker to selectively stain for injected human MSCs. We stained for PRSS36, CCR7, and SPARC and counted double positive cells (DP: hβ2M^+^/PRSS36^+^; hβ2M^+^/CCR7^+^; hβ2M^+^/SPARC^+^) as well as β2 microglobulin single positive MSCs. Intriguingly, we found that PRSS36, CCR7, and SPARC were significantly up-regulated in S100A8/A9 pre-treated MSCs after being injected to wounds compared to injected non-treated control MSCs. No expression of these proteins was found in PBS treated wounds. These data imply that the S100A8/A9 fingerprint is at least in part also true under *in vivo* conditions of S100A8/A9 primed MSCs injected into the wound site.

### Histological changes underlying accelerated wound healing in wounds injected with S100A8/A9 primed MSCs

Representative microphotographs depict H&E and Trichrome staining of PBS injected wounds, MSC injected wounds and wounds injected with S100A8/A9 primed MSCs at different time points after wounding (Supplementary Fig. 2 and 3). Intriguingly, reduced scab formation was observed in MSC injected wounds, and even less in wounds injected with S100A8/A9 primed wounds. Re-epithelialization occurs already at day 5 in wounds injected with S100A8/A9 primed MSCs, while MSC injected wounds show re-epithelialization only at day 10 after wounding. No re-epithelialization was at all observed in PBS injected control wounds at day 10 after wounding. The wound bed at day 10 is very large in PBS injected wounds (stippled line) as compared to MSC injected wounds and S100A8/A9 primed MSCs injected into wounds. Using Trichrome staining, there is almost no collagen deposition in the wound bed of PBS injected wounds (Supplementary Fig. 3 left panel). By contrast, at day 10 after wounding collagen deposition already occurred in MSC injected wounds (Supplementary Fig. 3 right panel) and in wounds injected with S100A8/A9 primed MSCs (Supplementary Fig. 3 lower left panel). Of note, wounds injected with S100A8/A9 primed MSCs depict collagen deposition which occurs in a wavy basket weaver fibrillary structure similar to the unwounded skin of the wound margin. By contrast, unprimed MSC injected wounds show a cell-rich dense collagen which in its structure is still distinct from that of unwounded skin at 10 days after wounding.

## Discussion

**Figure 7:**
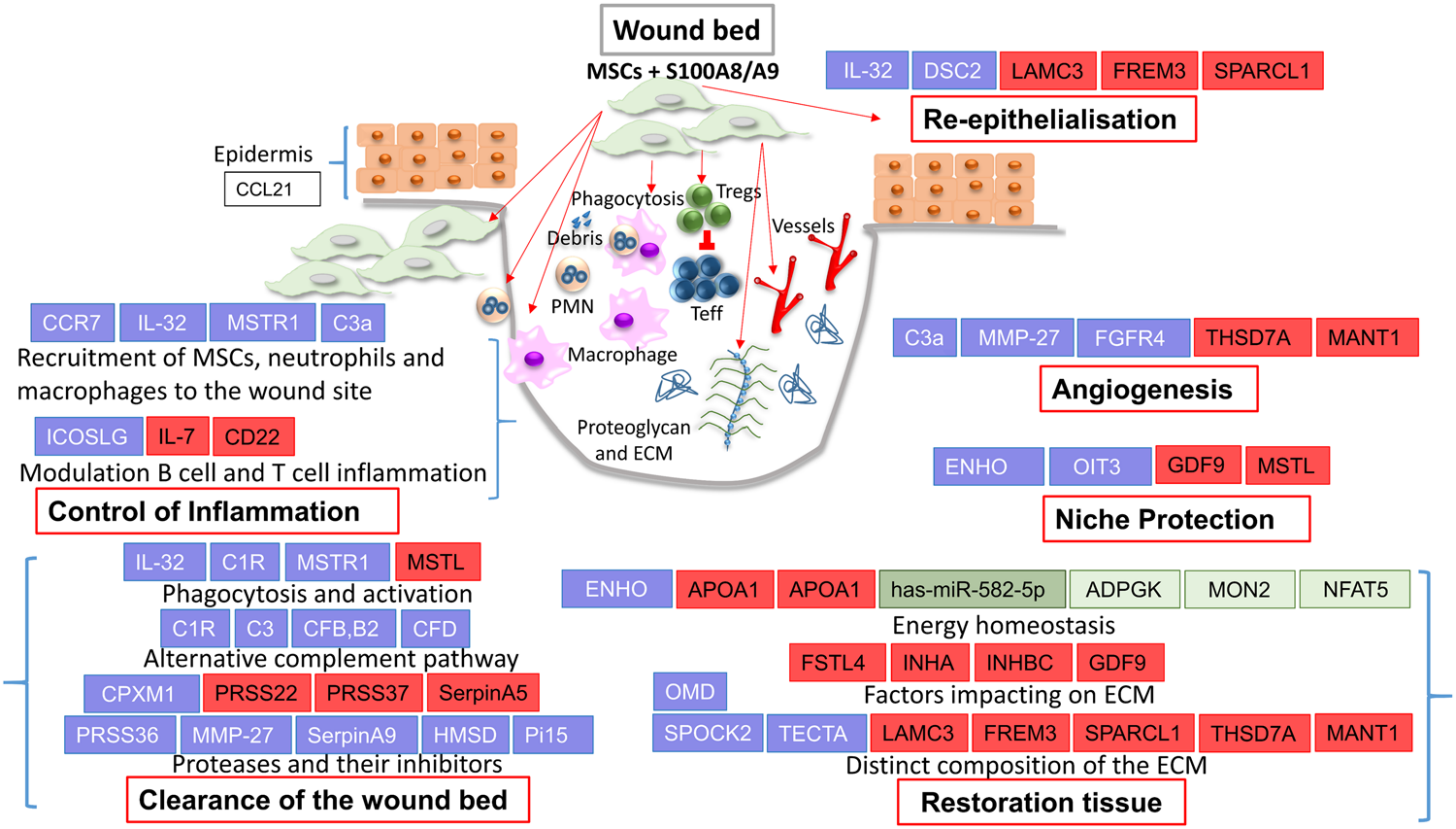
Graphical summary. Differentially regulated (DE) genes involved in specific wound healing phases (see red-rimmed boxes, e.g. control of inflammation, clearance of the wound bed, others) are depicted. The distinct wound healing phases are composed of several cellular functions (for example clearance the wound bed relies on proteases and their inhibitors, the alternate complement system and macrophage phagocytosis and activation). DE genes in MSCs treated with S100A8/A9 versus non-treated MSCs are depicted in blue (up-regulated) or red (downregulated). Differentially regulated genes are selected according to their known functions. Brackets aside indicate that genes and functions are related to a specific wound phase. Differentially up-regulated miRNAs are depicted in green and the corresponding target genes DE genes in MSCs treated with S100A8/A9 versus non-treated MSCs are depicted in blue (up-regulated) or red (downregulated). in light green. S100A8/A9 treated MSCs fundamentally switch their transcriptome to fast healing mode. Accordingly, the dramatic change in the transcriptome enforces a well-controlled influx of inflammatory cells which are predicted to be involved in the suppression of an unrestrained immune response and, importantly, in cleaning up the wound site (IL-32, MSTR1, C3a). In fact, activation and phagocytotic properties of macrophages are enhanced by the alternate complement system and activation of PI3 kinase upon binding of MSTR1 to its ligand. The debridement of the wound site is complemented by controlled activation of proteases and their inhibitors. At the same time, synthesis and deposition of a fetal repair like extracellular matrix (ECM) (e.g. SPARC encoded by SPOCK2) is promoted, while genes encoding distinct extracellular matrix proteins involved in scar formation are suppressed. This unique ECM signature is further implemented by the control of factors impacting on the proteoglycan rich ECM. ECM synthesis, in addition to angiogenesis, re-epithelialization and overall niche protection are highly energy consuming processes. Therefore energy homeostasis and the availability of sufficient ATP is a necessary request which according to the transcriptome is fully available. In consequence, accelerated tissue repair of wounds injected with S100A8/A9 treated MSCs is even better when compared to the already accelerated healing of wounds treated with control MSCs and, thus, holds substantial promise to be translated to clinical routine. The switch of the transcriptome of MSCs by danger molecules like S100A8/A9 may also be beneficial for the treatment of fibrotic conditions including hypertrophic scar formation. Teff, effector Tcells; Treg, regulatory T cells.

Our major finding is that pretreatment of MSCs with the DAMP protein S100A8/A9 and their subsequent injection into murine wounds results in significant acceleration of wound healing even exceeding enhanced healing of wounds injected with non-treated MSCs. This is an unexpected, previously unreported finding which holds substantial promise to refine and improve stem cell-based therapies for acute and chronic difficult-to-heal wounds. Employing an unbiased RNAseq approach, we uncovered a novel expression profile in S100A8/A9 treated MSCs with an impressive reprogramming of the transcriptome towards a protective niche which coordinates the function of inflammatory and resident cells at the wound site thus enhancing overall wound repair (Fig. 7, graphical summary). In the first instance, a network of genes involved in proteolysis and its control and genes enhancing macrophage phagocytosis profoundly clean up the wound site, and expression of genes involved in inflammation control and protection of resident stem cells. This expression profile reminiscent at least in part of scar-reduced tissue repair accelerates all wound healing phases in the interest of restoring tissue homeostasis after injury.

### Clearance of the wound bed and inflammation control

The adaptive response of MSCs to S100A8/A9 is unprecedented and the sequence of wound healing phases including inflammation, clearance of debris, regenerative tissue restoration with selected deposition of extracellular matrix, protection of the stem cell niche with energy homeostasis, angiogenesis and re-epithelialisation are not strictly separated, but according to the analysed transcriptome rather occur in parallel within a time frame of 18 h at least on the level of gene expression (summarized in Fig. 7). This underscores the concept that MSCs are endowed with the unique property to perfectly control multiple cell and tissue interactions like an orchestra conductor which coordinates all musicians in their complex interactions for the ultimate perfection of music. Accordingly, a gene signature serving tight inflammation control occurs after exposure of MSCs to the DAMP molecule S100A8/A9. This is initiated by the recruitment of MSCs to the wound site. Enhanced MSC recruitment occurs as a consequence of high expression of the gene coding for the CCR7 chemokine receptor after binding to its ligand CCL21 which is released from the wounded epithelium adjacent to the wound site^21,22^. Also, C3a recruits MSCs to the wound site^30^ and, importantly, enhances their survival at the wound site^31^. IL-32 initiates monocyte differentiation to phagocytically active macrophages^32^. IL-32 furthermore induces macrophage inflammatory protein-2 (MIP-2) and various chemokines and inflammatory cytokines, which themselves recruit neutrophils and macrophage to the wound site^33^. At the wound site macrophages together with resident MSCs^34^ initiate phagocytosis of tissue debris and apoptotic neutrophils. In fact, overexpression of macrophage stimulating receptor1 (MSTR1) on MSCs, a receptor normally expressed on phagocytically active macrophages, hints to the previously unreported enhanced phagocytotic activity of MSCs. The “big clean-up” after tissue trauma is further boosted by S100A8/A9 treated MSCs by the expression of several complement factors^35^ involved in the alternative complement pathway (summarized in Fig. 7) which via opsonization of damaged tissue by CFB activated C3b - additionally enhance the phagocytotic capacities of macrophages^32^. An important pillow of wound debridement and inflammation control is balanced proteolysis of damaged tissue. Balanced proteolysis most likely occurs by overexpression of serine proteases, matrix-metalloproteases and carboxypeptidases with distinct though not fully understood substrate specificities (PRSS36 coding for the polyserase-2^36^, MMP27 coding for the matrix-metalloprotease 27^37–39^, CPXM1 coding for the carboxypeptidase X, M14 Family Member 1^40^). By contrast, the expression of other serine protease family members are distinctly down-regulated (PRSS37^41^ and PRSS22^42^. Several other back-up inhibitors involved in the control of serine proteases are upregulated (Serpin A9^43^, HMSD^44^ and Pi15^45^). These back-up inhibitors belong to different families indicating the importance of a tight control of polymerase-2 and other serine protease activities in the wound bed. Given that we observed impressively reduced scab formation in wounds which were injected with S100A8/A9 primed MSCs, and polyserase-2 encoded by the PSSR36 gene, among other serine proteases, is up-regulated more than 10-fold in S100A8/A9 primed MSCs after injection into full-thickness wounds (Fig. 7 and Supplementary Fig. 2 and 3), we speculated that polyserase-2 may be involved in scab reduction. In fact, polyserase-2 is a secreted serine protease belonging to the family of S1 peptidases which are involved in fibrinolysis and complement activation. This may be very relevant in terms of reduced scab formation and, in consequence, improved wound healing. It may also be important for activation of complement-dependent phagocytosis (Fig. 7 and Supplementary Fig. 2 and 3). Interestingly, we also observed complement activation of the classical and the alternate pathways in wounds which have been injected with S100A8/A9 primed MSCs.

Inflammation control is also promoted by the up-regulation of the S100A8/A9 induced ICOSLG gene coding for the ligand for the T-cell-specific cell surface receptor ICOS. ICOS belongs to the CD28 co-stimulatory molecule family. Hence, it acts as a co-stimulatory signal for T-cell proliferation^23,24^, skewing cytokine secretion towards a Th2 cell helper type with enhanced release of anti-inflammatory molecules like IL-4^25^ IL-4 itself promotes an anti-inflammatory M2 macrophage which is essential for proper wound repair^3^. Of note, enhanced expression of ICOSLG on S100A8/A9 induced MSCs also drives the activation of regulatory T cells^46^. This is highly interesting as regulatory T cells play an important role in re-epithelialization and wound closure^47^. The S100A8/A9 induced ICOS ligand in MSCs is critical to mediate local protective tissue responses to injury and inflammatory conditions. Less obvious is the role of reduced CD22 expression on S100A8/A9 induced MSCs. CD22 is a cell surface molecule of the differentiation cluster which so far is considered to be restricted to B-cells and its activation^48^.

### Induction of restoration tissue, angiogenesis, and re-epithelialization

Remarkably, for the benefit of wound healing S100A8/A9 increase the expression of a unique combination of extracellular matrix coding genes (Spock-2, TECTA, OMD) in MSCs, while suppressing others (LAMC3, FREM3, SPARCL1, THSD7A, and MANT1). In this regard, the SPOCK-2 gene encodes a glycoprotein (SPARC, BM-40, Osteonectin) which binds to glycosaminoglycans as an important core component of proteoglycans in the extracellular matrix. Increase in the SPOCK-2 encoded SPARC expression occurs in actively proliferating cells and organs such as developing embryos and adult tissues related to tissue remodeling, wound healing and angiogenesis (reviewed in^49,50^). SPARC mediates cell-matrix and matrix-matrix interactions, and thus organizes the supramolecular structure of the restoration tissue. The importance of SPARC becomes clear in SPOCK-2 deficient mice which display severely delayed wound healing^51^. SPARC also serves as a reservoir for growth factors. Among others, platelet derived growth factor (PDGF-BB) which stimulates granulation tissue formation and angiogenesis and constitutes an important survival signal for MSCs in their niche^52,53^ binds to SPARC. The TECTA gene codes for α-tectorin is significantly induced in S100A8/A9 treated MSCs. α-tectorin is a major non-collagenous connective tissue component of the tectorial membrane in the inner ear. Mutations in the TECTA gene have been reported to be responsible for autosomal dominant non-syndromic hearing impairment and a recessively inherited disorder of sensorineural pre-lingual non-syndromic deafness^54,55^. It is most interesting that TECTA which - to the best of our knowledge - has not been described in MSCs nor in the skin is induced in S100A8/9 treated MSCs. Its function in wounding is unclear, but similarly to SPARC, it may contribute to a provisional restoration matrix which promotes tissue repair. OMD coding for osteomodulin, a keratan sulfate-rich proteoglycan, is up-regulated in S100A8/A9 treated MSCs. Osteomodulin binds to the α_v_β_3_ integrin^56^ and is involved in organizing the extracellular matrix. Interestingly, recombinant osteomodulin suppressed collagen type I fibril formation^57^. As unrestrained synthesis and collagen type I fibril deposition is a hallmark of scars, osteomodulin may exert a previously unknown antifibrotic function. Of note, we observed a qualitatively much better extracellular matrix structure reminiscent of unwounded, normal skin. In fact, trichrome staining indicative of collagen deposition and structure, shows a wavy basket weaver fibrillary structure of collagen in wounds injected with S100A8/A9 primed MSCs. This structure is similar to the unwounded skin (Supplementary Fig. 3).

Many extracellular matrix proteins are suppressed following exposure of MSCs to S100A8/A9, mainly those which inhibit cell migration during re-epithelialization and angiogenesis. This most likely applies for LAMC3 coding for the gamma 3 chain of laminin12, a multidomain cruciform protein within the skin^58,59^, FREM3 coding for FRAS1 related matrix protein 3 which Ca^+^- dependently ensures the 3-dimensional structure of the basement membrane of the dermo-epidermal junction^60^. Similarly, SPARC like protein 1 (SPARCL1)^61^ suppresses cell migration^62^. thrombospondin (THSD7A) and matrilin-1 (MANT1) inhibit angiogenesis^63,64^. Also, growth differentiation factor GDF9 normally highly expressed in fibroproliferative conditions as in keloids and hypertrophic scars^65^ is significantly reduced further underscoring the antifibrotic signature of the transcriptome of S100A8/A9 treated MSCs. These findings are particularly important as we found a substantial increase in re-epithelialization in wounds injected with S100A8/A9 primed MSCs as opposed to PBS injected wounds or MSC injected wounds (Supplementary Fig. 2 and 3).

Several factors down-regulated by S100A8/A9 pretreatment of MSCs may enhance wound healing. Physiologically, activin enhances wound healing by activating both fibroblasts and keratinocytes thus promoting restoration tissue formation and re-epithelialisation^66^. In case of S100A8/A9 pretreated MSCs inhibitors of activin (FSTR4, a gene encoding Follistatin-related protein 4, INHA and INHBC coding 2 types of inhibins) are significantly suppressed, and this, in consequence, may result in a net enhancement of activin function and accelerated tissue repair.

### Energy Homeostasis

Wound healing is an energetically highly demanding process. This includes all phases in particular matrix synthesis and its supramolecular highly organized deposition. The S100A8/A9 induced MSC transcriptome includes genes (ENHO, APOC2, APOA1) and also miR 582-5p with the suppression of 3 energy consuming target genes, all together boosting energy supplying pathways. This energy boosting transcriptome of S100A8/A9 treated MSCs is distinctly different from non-treated control MSCs and, most likely, is beneficial for tissue repair. In this regard, ENHO (energy homeostasis associated) is a protein-coding gene. It is involved in the regulation of glucose homeostasis and lipid metabolism. In addition, ENHO and its encoded protein product adropin protect endothelial cells and most likely also MSCs and other resident stem cells from oxidative damage via activation of the endothelial nitric oxide synthase and the PI3K-Akt pathway^67,68^. ENHO mutations with dysfunctional Adropin, in fact, constitute a major risk factor in the development and severity of the ANCA associated vasculitis (inflammation of vessels)^69^. ApoliproteinA1 (APOA1) and apolipoprotein C2 (APOC2) are major lipid binding proteins and physiologically bind lipids and cholesterol. Both genes coding for apolipoproteins are significantly down-regulated in S100A8/A9 treated MSCs. Among several possibilities, this may imply that unbound lipids and cholesterol can be channeled into energy producing pathways like β-oxidation and with the resulting AcylCoA which thereafter serves for ATP generation in the citrate cycle^70^. Finally, miR 582-5p is significantly induced in S100A8/A9 treated MSCs. miR 582-5p is predicted to bind to the 5´UTR region and inhibit corresponding target genes which drive energy consuming processes. The miR 582-5p suppressed genes like the ADPGK codes for the human ADP-dependent glucokinase which is involved in glycolysis^26^. Glycolysis is energetically less favorable in terms of ATP output when compared to the citrate cycle. The miR 582-5p suppressed human MON2 gene encodes a protein which controls the energy demanding traffic between endosomes and the Golgi. Finally, miR 582-5p suppresses the NFAT5 gene. NFAT induces osteogenic differentiation of MSCs^27^ which, in fact, would demand a lot of energy, and is distinctly counterproductive for cutaneous tissue repair.

### Niche homeostasis

Boosted energy homeostasis, activation of the PI3 kinase growth and other survival pathways (C3^30,31^, Adropin/ENHO^67,68^, Oncogene induced transcript 3 protein/OIT3^71^, MSTR1^72,73^) a specific MSC imprinted environment including inflammation control, cleaning up tissue debris and danger associated molecular patterns (see Fig. 7 and above), enhancing nutrition and oxygen supplying angiogenesis, and growth factor binding extracellular matrix (SPARC encoded by SPOCK-2, others see Fig. 7), collectively encraft a beneficial niche which protects MSCs and resident stem cells from severe damage and in consequence enable S100A8/A9 MSCs to orchestrate wound healing at impressively accelerated speed.

In aggregate, we here uncovered that MSCs when exposed to the danger signal molecule S100A8/A9 raise a beneficial adaptive response with accelerated wound healing. The underlying transcriptome signature is novel and, importantly, extends the previously coined MSC property of a “drug store”^74^ to the clinically most relevant “adaptive drug store” which is advantageous over the delivery of defined recombinant factors in that MSCs can sense their environment and within short time adapt their transcriptome for the sake of tissue renewal and homeostasis. It is possible that other mechanisms of triggering activation of the MSCs also enhance wound healing, and thus would support the idea of an “adaptive drug store”. This may hold for LPS, a wall constituent of gram-negative bacteria, which acts through the same TLR4 receptor. It will be interesting to see whether this, in fact, is the case, and if so whether an identical or a clearly distinct expression profile is induced by LPS as opposed to S100A8/A9.”

This novel concept is highly suitable to be introduced into the clinical application to treat acute and difficult-to-treat wounds and most likely other fibroproliferative disorders like hypertrophic scars or fibrotic diseases of internal organs.

## Methods

### Adipose tissue derived mesenchymal stem cells

Human adipose tissue-derived mesenchymal stem cells (MSCs) at passage 1 were purchased from A.T.C.C. (# ATCC-PCS-500, LGC Standards GmbH, Wesel Germany) and grown at 5% CO_2_ and 37 °C in Mesenchymal Stem Cell Basal medium supplemented with low serum growth kit (ATCC-PCS-500-030 and ATCC-PCS-500-040). All MSCs employed for experiments were below passage 3. FITC or PE or APC conjugated anti-human antibodies CD73, CD90, CD105, CD14, and CD34 were purchased from MSC phenotyping kit, human (Cat # 130-095-198, Miltenyi Biotech GmbH, Germany). AT-MSCs were harvested, washed with PBS, and incubated with antigen-specific antibodies for 15 min at 4 °C in dark. The non-specific staining was controlled by isotype-matched antibodies for the corresponding fluorochrome channels. Flow cytometry was performed on an LSRFortessa (BD Biosciences, CA, USA) with FACSDiva software for data acquisition (BD Biosciences). Data were analyzed with FlowJo (FlowJo, LLC, USA).

### Adipogenic, osteogenic and chondrogenic differentiation of MSCs

The induction of adipogenic, osteogenic and chondrogenic differentiation of MSCs and the subsequent Oil-Red-O staining, Alizarin-Red-S staining, and immunostaining with a specific anti-aggrecan human antibody was performed as described previously^75^.

### MSC treatment with S100A8/A9

MSCs were grown in MSCs media (#ATCC-PCS-500-030) till 80% confluency. 10^6^ cells were plated per flask and cultured for either 6 or 24 hours either in MSC media or MSC media supplemented with 0.5 µg/mL of Rh S100A8/A9 (R&D Systems). Thereafter, S100A8/A9 primed and unprimed MSCs were harvested and subjected to RNA sequencing or for injection into the wound margin of murine full thickness wounds.

### RNA isolation

Isolation of mRNA was performed from MSCs which had been exposed to 0.5 µg/ml S100A8/A9 for 6 and 24 h, respectively. Thereafter, MSCs were flushed with 10 ml PBS and scraped from cell culture plates on ice. Cell pellets were incubated with 100 µl of RNA later (Ambion, Cat # AM7020). Total RNA was extracted from MSC using Qiagen reagent (Life Technologies, Grand Island, NY, USA) according to the manufacturer’s instructions and stored at −80 °C until further analysis. RNA quality was verified with Bioanalyzer and RNA samples were considered for further analysis when showing RIN number between 9 and 10. Control mRNA samples were isolated from non-treated MSCs. The miRNA was isolated with miRNA isolation kit (Roche, Cat #05080576001) as suggested by the manufacturer´s instructions using a 2 step isolation protocol. The miRNA quantification was carried out using Qubit 3.0 Fluorometer and Quant-IT miRNA assay kit (Thermofisher Scientific, Cat # Q32882).

#### RNA-seq library preparation and sequencing

Illumina mRNA-seq libraries were prepared with the TruSeq RNA kit (version 1, Rev A), using 1 μg of total RNA according to the manufacturer’s instructions. For HiSeq. 2000 sequencing, eight libraries were pooled per sequencing lane. The pooled library samples were sequenced on the Illumina Hiseq. 2000. The raw data from the sequencing run is initially assessed for Illumina low Phred base score and the presence of adapter sequences using the FastQC program. The phred score of Q30 was obtained for all the FastQ RNAseq Files. Following initial analysis, reads that passed the QC were processed using the New Tuxedo Algorithm^76,77^. Briefly, RNAseq reads were aligned to the Human reference genome hg38 annotation using the HISAT2 algorithm and read counting overlapping gene counting overlapping gene features was done using HTseq. Mapping efficiency and quality were assessed using the RNAseq software. The number of reads mapping to annotated gene features was counted using HTSeq using an hg38 General Feature Format (GTF) annotation file. The resulting counts were imported to R and the Bioconductor package DESeq. 2 was used for differential gene expression analysis.

The Nucleotide Sequences are archived at European Nucleotide Archive (ENA) with accession number PRJEB23139 as primary accession number and ERP104872 as secondary accession number. https://www.ebi.ac.uk/ena/data/view/PRJEB23139.

#### RNAseq expression visualization

Fold change of differentially regulated genes and their adjusted p-values were depicted using volcano plot using the R package.

#### Pathway Impact analysis

The pathway plot was analyzed via iPathway Guide using impact analysis that looks at over-representation of differentially expressed genes in a given pathway and the total gene accumulation in a given pathway^78^. The underlying pathway topologies, comprised of genes and their directional interactions, are obtained from the KEGG database^79^.

### Hierarchical clustering

The hierarchical clustering and Venn plot were carried out using rpudplus, hclust and Venn diagram function in R-Studio employing differentially expressed pre-ranked genes which were associated with extracellular secretory function as classified using TopGO R-Studio package and GeneTrail2. The principle component analysis (PCA) based on regularized log transformed FPKM values with an adjusted p-value < 0.05. obtained from DEseq. 2 and was carried out using ggfortify and ggplot2 packages in R-Studio. (R Tutorial with Bayesian Statistics Using OpenBUGS, Chi Yau, ASIN: B006ZP4SKW).

#### RNA extraction and quantitative reverse transcriptase/real time-PCR (qRT-PCR)

To validate the RNA-Seq data, we performed qRT-PCR of selected genes enrolling MSCs treated with S100A8/A9 for 6 and 24 h, respectively as opposed to untreated MSCs. RNA was isolated as described above. The AD-MSC were subjected to M-MLV reverse transcriptase RNase H Minus (Promega, #M3681) and Oligo dT primers 0.5 µg/µl (Promega, #C1101) to generate complementary DNA and the miRNA cDNA was transcribed using miScript II RT kit (Qiagen, Cat # 218160), that was diluted to 4 ng RNA/well. The cDNA was further amplified with gene-specific primers 10 pMol (Supplementary Tab. 1) and SYBR Green 2× master mix (Applied Biosystem, # 4309155) for (95 °C, 5 min) denaturation, [(95 °C, 1 min), (60 °C, 1 min), (72 °C, 1 min)] amplification qPCR cycle. The qPCR data were normalized with β-actin whereas miRNA gene expression was normalized with RNAU6B. Statistical analysis was done using paired, two-tailed Student’s t-test followed by Wilcoxon matched pairs signed rank test. P values were assigned * with p < 0.05, **p < 0.01, *** < 0.001 and ****p < 0.0001.

#### Mice and wound-healing model

All experiments were performed with 6-week-old C57BL/6 mice in compliance with the German Law for Welfare of Laboratory Animals and were approved by the Institutional Review Board of the University of Ulm. Prior to the injury, mice were anesthetized by intraperitoneal injection of a ketamine (10 g/l)/xylazine (8 g/l) solution (10 μl/g body weight). After shaving the dorsal hair and cleaning the exposed skin with 70% ethanol, full‐thickness (including the *panniculus carnosus*) excisional wounds were punched at two sites in the middle of the dorsum using 6‐mm round knives (STIEFEL, Offenbach, Germany). Each wound region was digitally photographed at indicated time points, and wound areas were calculated using Photoshop^®^ software (version 7.0; Adobe Systems, San Jose, CA)^3^. The wound size at any given time point after wounding was expressed as a percentage of initial (day 0) wound area. Wounds were harvested for immunostaining at the indicated time points.

### Immunostaining of wound sections

Immunostaining of sections from paraffin-embedded wounds was performed using a previously described protocol^28^. Paraffin sections of untreated control wounds, wounds injected with untreated MSCs, and MSC pretreated with S100A8/A9 were incubated with antibodies against anti-β_2_microglobulin (Abcam Cat #ab175031) anti-CCR7 (Abcam Cat #32527), anti-PRSS36 (Sigma-Aldrich Cat # HPA036079), anti-SPARC (R&D system, Cat #MAB2328) Isotype IgG served as negative control. Photomicrographs were taken using a Zeiss Axiophot microscope and AxioVison 4.8 software (Zeiss).

### Trichrome and H&E staining of wound sections

These techniques were performed as previously described^7^.

### Statistical analysis

Data were analyzed with GraphPad Prism software (GraphPad Software, Inc., San Diego, CA). For correlation between control wounds, wound injected with untreated MSCs and with S100A8/A9 treated MSCs, multiple groups were analyzed for significance using ANOVA with non-parametric measures using Kruskal Wallis and Dunn’s post hoc test. Non-parametric qPCR data were analyzed using the paired, two-tailed non-parametric test using Wilcoxon signed rank-test. Parametric one-way analysis of variance was used with Tukey’s posthoc analysis for comparison of cell counts taken from multiple groups representative photomicrographs. Student’s *t*-test was used for comparison between two groups. Significance was defined as *P* < 0.05. P values were assigned * with P < 0.05, ** with P < 0.01, *** with P < 0.001 and **** with P < 0.0001.

## Electronic supplementary material

Supplementary Figures
