# Author Correction: A Novel S100A8/A9 Induced Fingerprint of Mesenchymal Stem Cells associated with Enhanced Wound Healing

**DOI:** 10.1038/s41598-018-28097-3

**Published:** 2018-07-02

**Authors:** Abhijit Basu, Saira Munir, Medanie A. Mulaw, Karmveer Singh, Diana Crisan, Anca Sindrilaru, Nicolai Treiber, Meinhard Wlaschek, Markus Huber-Lang, Florian Gebhard, Karin Scharffetter-Kochanek

**Affiliations:** 1 Experimental Laboratories of the Department of Dermatology and Allergic Diseases, Ulm University, Life Science Building N27, James-Franck-Ring, 89081 Ulm, Germany; 2 Institute of Experimental Cancer Research, Ulm University, Life Science Building - N27, James-Franck-Ring, 89081 Ulm, Germany; 3 Department of Dermatology and Allergic Diseases, Ulm University, Albert-Einstein-Allee 23, 89081 Ulm, Germany; 4 Institute for Clinical and Experimental Trauma Immunology (ITI), Ulm University, Helmholtzstraße 8/2, 89081 Ulm, Germany; 5 Department of Orthopaedic Trauma-, Hand-, Plastic, and Reconstruction Surgery, Ulm University, Albert-Einstein-Allee 23, 89081 Ulm, Germany

Correction to: *Scientific Reports* 10.1038/s41598-018-24425-9, published online 18 April 2018

The original version of this Article contained errors.

Affiliations 1, 2, 3, 4 and 5 were incorrectly listed as ‘Experimental Laboratories of the Department of Dermatology and Allergic Diseases, University of Ulm, Life Science Building N27, James-Frank-Ring, 89081, Ulm, Germany’, ‘Institute of Experimental Cancer Research, University of Ulm, N27 Life Science Building, James Frank-Ring, 89081, Ulm, Germany’, ‘Institute for Clinical and Experimental Trauma Immunology (ITI), Ulm University, Helmholtzstraße 8/1, 89081, Ulm, Germany’, ‘Department of Orthopaedic Trauma-, Hand-, Plastic, and Reconstruction Surgery, Ulm University, Albert-Einstein-Allee 23, 89081, Ulm, Germany’ and ‘Department of Dermatology and Allergic Diseases, Ulm University, Albert-Einstein Allee 23, 89081, Ulm, Germany’ respectively. The correct affiliations are listed below.

Affiliation 1:

Experimental Laboratories of the Department of Dermatology and Allergic Diseases, Ulm University, Life Science Building N27, James-Franck-Ring, 89081 Ulm, Germany

Affiliation 2:

Institute of Experimental Cancer Research, Ulm University, Life Science Building - N27, James-Franck-Ring, 89081 Ulm, Germany

Affiliation 3:

Department of Dermatology and Allergic Diseases, Ulm University, Albert-Einstein-Allee 23, 89081 Ulm, Germany

Affiliation 4:

Institute for Clinical and Experimental Trauma Immunology (ITI), Ulm University, Helmholtzstraße 8/2, 89081 Ulm, Germany

Affiliation 5:

Department of Orthopaedic Trauma-, Hand-, Plastic, and Reconstruction Surgery, Ulm University, Albert-Einstein-Allee 23, 89081 Ulm, Germany

Additionally, Benedikt Herold, who had been included as an author on the original version of the Article, did not contribute sufficiently to be listed as an author. He has therefore been removed from the author list.

Finally, in the original version of this Article Abhijit Basu and Saira Munir were omitted as equally contributing authors. The author contribution section now reads:

“A.B. and S.M. contributed equally to the study design, conducted the experiments, analyzed the results and wrote parts of the manuscript. M.A.M. supported this manuscript by his biometric expertise. K.S. contributed to the study design and performed qRT-PCR experiments. D.C., A.S., N.T., M. H.-L., F.G., and M.W. contributed to the discussion, design and the interpretation of the data in the context of trauma and wound healing. K.S.-K. designed the experiments and wrote the manuscript.”

These errors have now been corrected in the PDF and HTML versions of the paper, and in the accompanying Supplementary Figures file.

