# Supplementary material for: A Novel S100A8/A9 Induced Fingerprint of Mesenchymal Stem Cells associated with Enhanced Wound Healing
Source: Sci Rep. 2018 Apr 18;8:6205. doi: 10.1038/s41598-018-24425-9 (PMC5906602; doi:10.1038/s41598-018-24425-9)
Supplement: Supplementary file 1 — Supplementary Figures [file 41598_2018_24425_MOESM1_ESM.pdf]

A Novel S100A8/A9 Induced Fingerprint of Mesenchymal Stem Cells associated with Enhanced Wound Healing

Abhijit Basu[1*], Saira Munir[1*], Medanie A. Mulaw[2], Karmveer Singh[1], Diana Crisan[1,3], Anca Sindrilaru[1,3], Nicolai Treiber[1,3], Meinhard Wlaschek[1], Markus Huber-Lang[4], Florian Gebhard[5] and Karin Scharffetter-Kochanek[1,3]

Short Title: The S100A8/9 Fingerprint of Mesenchymal Stem Cells

[1] Experimental Laboratories of the Department of Dermatology and Allergic Diseases, Ulm University, Life Science Building N27, James-Franck-Ring, 89081 Ulm, Germany

[2] Institute of Experimental Cancer Research, Ulm University, Life Science Building N27, James Franck-Ring, 89081 Ulm, Germany

[3] Department of Dermatology and Allergic Diseases, Ulm University, Albert-Einstein-Allee 23, 89081 Ulm, Germany

[4] Institute for Clinical and Experimental Trauma Immunology (ITI), Ulm University, Helmholtzstraße 8/2, 89081 Ulm, Germany

[5] Department of Orthopaedic Trauma-, Hand-, Plastic, and Reconstruction Surgery, Ulm University, Albert-Einstein-Allee 23, 89081 Ulm, Germany

*Contributed equally

Supplementary Figure. 1

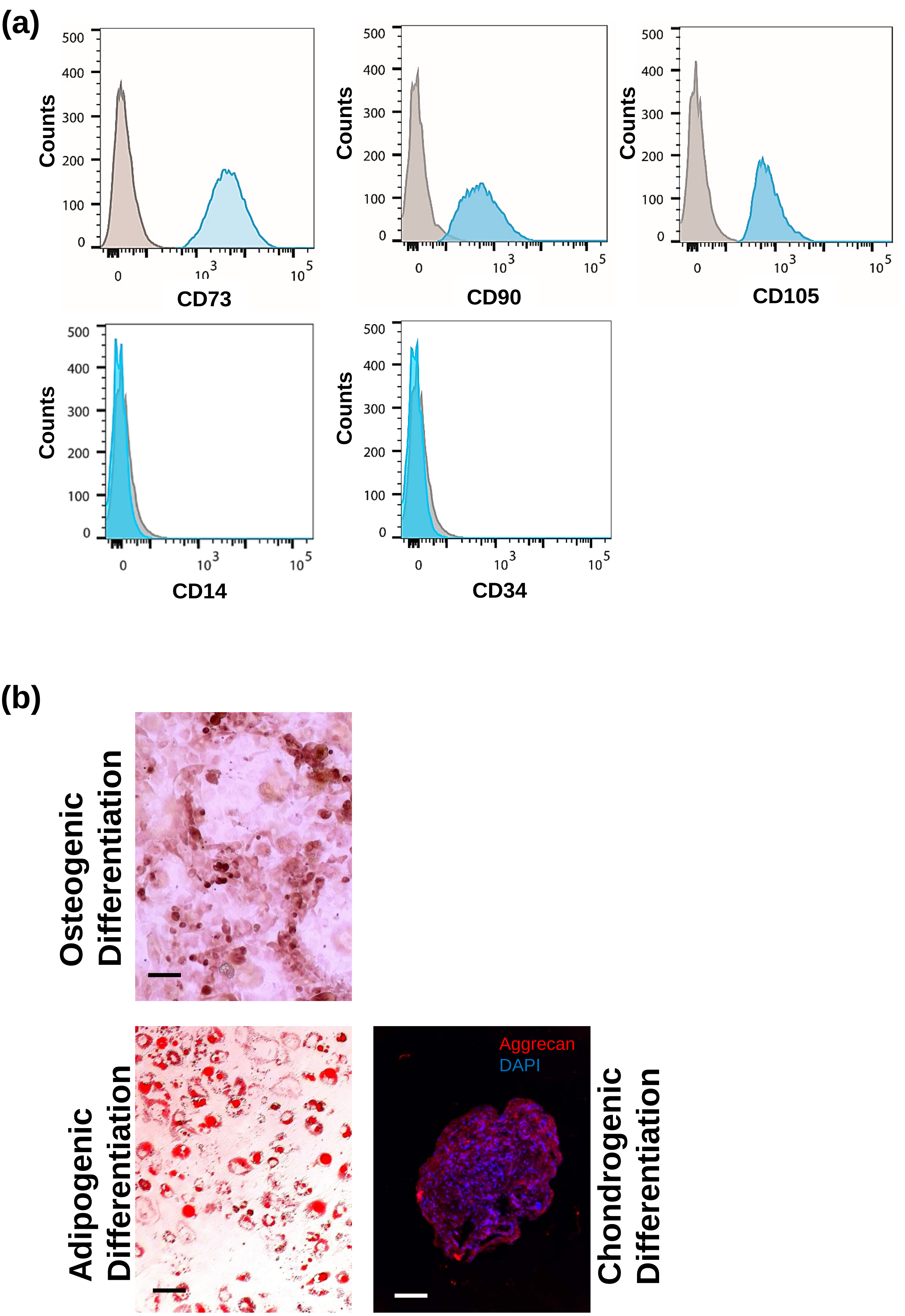

Supplementary Figure. 1 (**a**) Expression of surface markers of AT-MSC was analysed by flow cytometry, including CD73, CD90, CD105, CD14 and CD34. Stem cell specific surface marker expression on MSCs were highlighted by blue histograms and the isotype control stained MSCs were highlighted by grey histograms. (**b**) 10^5 MSCs were seeded and were cultured in adipogenic induction, osteogenic induction and chondrogenic induction medium for 21 days. The cells were fixed with PFA and stained with Oil-Red-O staining indicative for adipogenic differentiation and with Alizarin-Red-S for osteogenic differentiation. An antibody against human aggregan was used to stain for MSCs with chondrogenic differentiation. Scale bar 200 µM

Supplementary Figure 2.

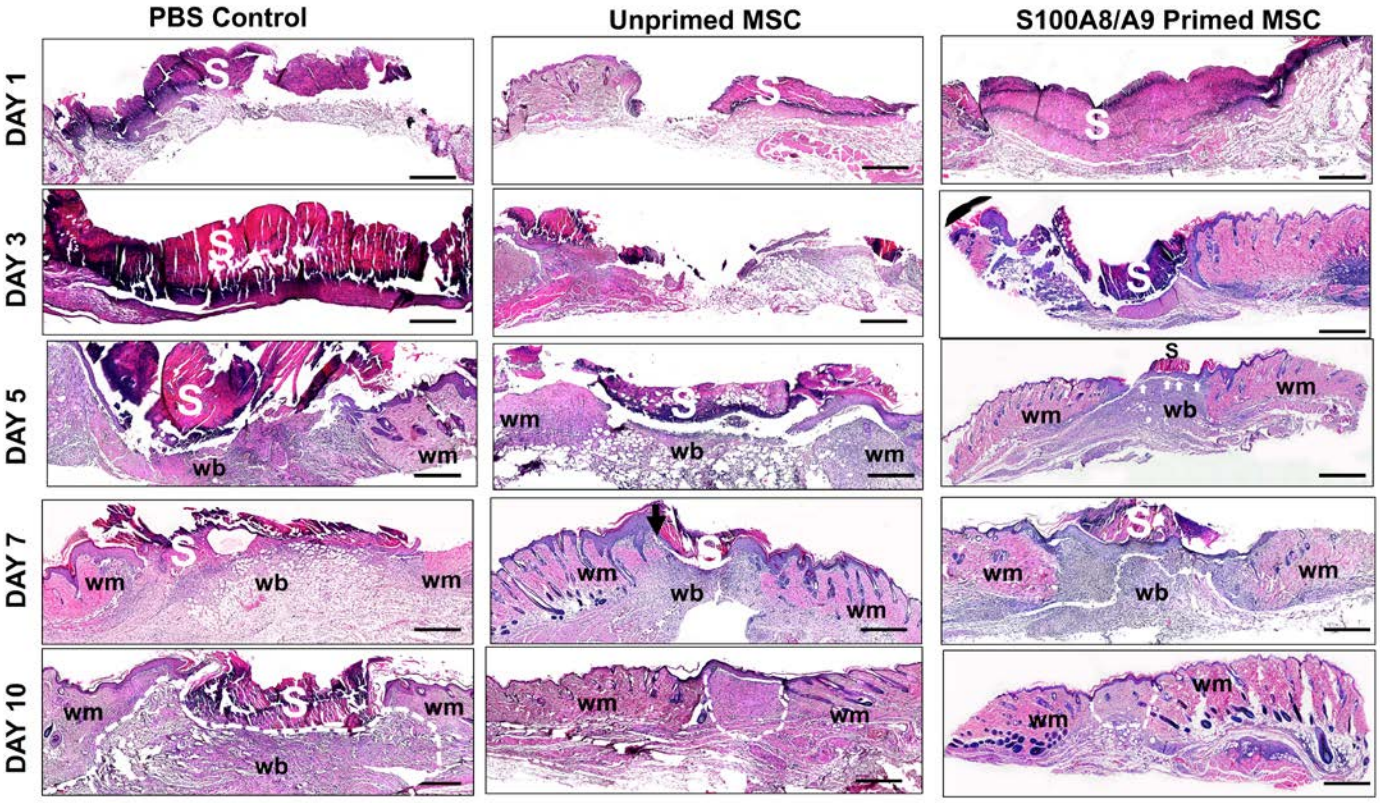

Supplementary Figure 2. Injection S00A8/A9 of primed MSCs into wounds result in acceleration of wound healing.

Representative microphotographs depict H&E staining of PBS injected wounds, MSC injected wounds and wounds injected with S100A8/A9 primed MSCs at different time points after wounding. Of note, there is reduced scab formation (S) in MSC injected wounds, and even less in wounds injected with S100A8/A9 primed wounds. Re-epithelialisation occurs already at day 5 in wounds injected with S100A8/A9 primed MSCs (white arrows), while MSC injected wounds show re-epithelialisation only at day 10. No re-epithelialisation was observed in PBS injected control wounds at day 10 after wounding. Wound area at day 10 is very large in PBS injected wounds (stippled line) as compared to MSC injected wounds and S100A8/A9 primed MSCs injected into wounds. S Scab, wb wound bed, wm wound margin. Scale bars: 200 µm.

Supplementary Figure 3.

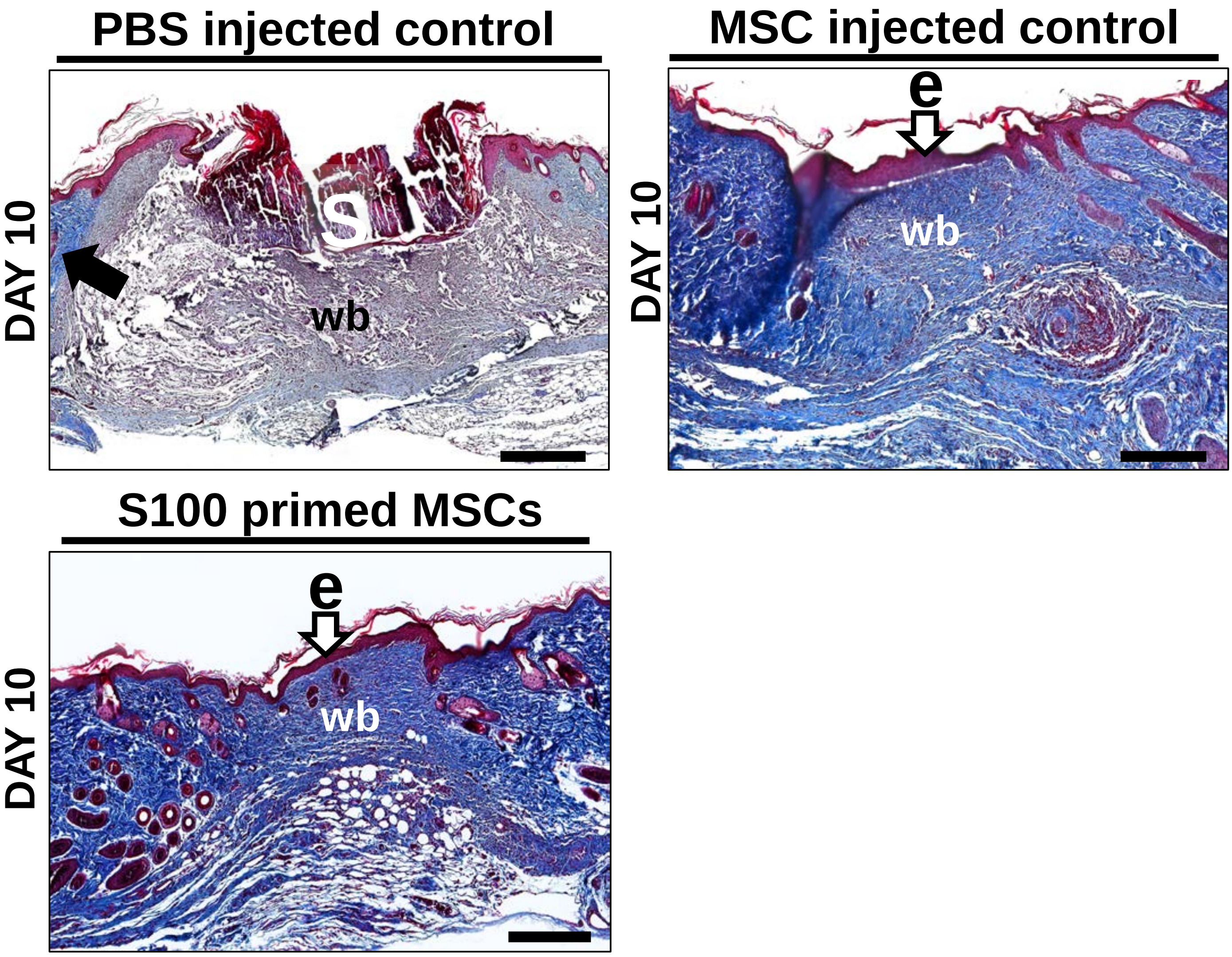

Supplementary Figure 3. Injection of S100 primed MSCs into wounds reveals a faster deposition of collagen mimicking unwounded skin.

Representative photomicrographs depict Trichrome stained sections of full thickness wounds at day 10 after wounding. Note that the PBS injected control wound (left top panel) do not depict blue staining in the wound bed (wb) as compared to the blue staining (collagen deposition) of the wound margin (↑). This indicates that there is almost no collagen deposition in the wound bed. By contrast, at day 10 after wounding collagen deposition already occurred in MSC injected wounds (right top panel) and in wounds injected with S100A8/A9 primed MSCs (lower left panel). Of note, wounds injected with S100A8/A9 primed MSCs depict a collagen depostion which is in a wavy basket weaver structure similar to unwounded skin of the wound margin. By contrast, unprimed MSC injected wounds show a cell rich dense collagen which in ist structure is still distinct from that of unwounded skin at 10 days after wounding. Please note that there is still severe scab formation (S) without any reepithelilsation in PBS injected control wounds as opposed to no scab formation in MSC and S100 A8/A9 primed MSCs which were injected into wounds. While MSCs injected wounds still depict a thickening of the epidermis, the reepithelised epidermis in S100A8/A9 primed MSCs which had been injected into wounds have an almost normal thickness of the epidermis. S scab, e epidermis, wb wound bed. Scale bars: 20µm.

Supplementary Tab. 1

Gene	5' End	3' End
MMP27	GCCAATTCATGCCACGTCTC	AAGCCGGTTCTGTTAGCACA
DSCC2	CATCTGCGGAGATTGTTGCG	AAAGTCAAAGGGTGGGCCAT
SPOCK2	GTGAGAGGTTTGGGGAGGTG	TCCATGGAGTTTCACGGTCG
PRSS36	TCCGCTGCTCACTGTTTCAT	CAGGACCAGGGCGAATACTC
ICOSLG	CATTGGCTGCTGCATAGA	GGATTCTCTGTGATCTTGTCTC
CCR7	TGTGGGCATCTGGATACT	CCACCTGGATGGTGATAAAG
OIT3	CCTAGATCCTTGTTCTGCTTAC	CATGGTTGTCACATAGAGGAG
IL-32	CTTTCTGAGTGTCACCGTTATT	AGAAGTAGGGAGGAGCATTAC
SERPINA9	CCACCACCAAGTTCATAGTC	ATACCGTCTGTGGCTTTATTT
PI15	GACCATGGACCTTCTTACTTAC	CTTGACCAACTGGAGAATAGAG
β-Actin	TGAGAGGGAAATCGTGCGTG	TGCTTGCTGATCCACATCTGC
Mir-582-5p	UAACUGGUUGAACAACUGAACC	
RNAUB	GTTGGCTCTGGTGCAGGGTCCGA GGTATTCGCACCAGAGCCAACAA AAATAT	

Supplementary Table. 1 Primer sequences used to quantify the mRNA through real time qPCR
